# Intraguild Interactions between Two Egg Parasitoids of a True Bug in Semi-Field and Field Conditions

**DOI:** 10.1371/journal.pone.0099876

**Published:** 2014-06-18

**Authors:** Ezio Peri, Antonino Cusumano, Valentina Amodeo, Eric Wajnberg, Stefano Colazza

**Affiliations:** 1 Dipartimento di Scienze Agrarie e Forestali, Università degli Studi di Palermo, Palermo, Italy; 2 INRA, Sophia Antipolis Cedex, France; Rutgers University, United States of America

## Abstract

Research on interspecific competitive interactions among insect parasitoids has often been characterized by laboratory studies in which host insects are exposed to female parasitoids of different species in various sequences and combinations. In the last years, an increasing number of studies have investigated interspecific interactions under field and semi-field conditions although just a few number of works focused on egg parasitoids. In this work, we undertook a two-year study to investigate interspecific interactions between *Trissolcus basalis* (Wollaston) (Hymenoptera: Platygastridae) and *Ooencyrtus telenomicida* (Vassiliev) (Hymenoptera: Encyrtidae), two egg parasitoids of the pest *Nezara viridula* (L.) (Heteroptera: Pentatomidae) that co-occur in cultivated crops. Under semi-field (in out-door mesh cages) and field conditions, we investigated: 1) the seasonal occurrence of competing parasitoid species on sentinel egg masses; 2) the impact achieved by competing species on the shared host on naturally laid egg masses; 3) the outcome of intraguild interactions under controlled conditions. Results from sentinel egg masses showed that *T. basalis* occurs in May and successfully parasitizes hosts until the end of September/beginning of October, whereas *O. telenomicida* is mainly occurring in July-August. In both years, it was found that *T. basalis* is predominant. From naturally laid egg masses, results indicated that *T. basalis* achieves higher impact on the hosts, even in those egg masses which are parasitized by more than one female of different species ( =  multiparasitism). Results from manipulating intraguild interactions showed that *T. basalis* achieves higher impact on *N. viridula* when released alone, but it suffers from competition with *O. telenomicida*. The ecological factors that play a role in intraguild interactions in the context of biological control perspective are discussed.

## Introduction

During the host location process, parasitoids may experience complex interactions with other parasitoids, hyperparasitoids, predators, and entomopathogens [1]–[3]. There are many examples where a single insect host suffers attack from a range of parasitoid species [4]. Intraguild interactions between parasitoids can play an important role in species coexistence, in shaping community structures and can have important consequences for biological pest control [5]. When parasitoid species compete for the same host ( =  interspecific competitive interactions) the interactions can be divided into two broad categories: the interactions that occur among adult females searching for or exploiting hosts ( =  extrinsic competition) and the interactions that occur among supernumerary larvae developing in the same host ( =  intrinsic competition) [6], [7].

In the context of biological control of phytophagous pests attacking crops, a key limiting aspect in understanding how interspecific competition affects pest suppression is the lack of experimental data from controlled experiments carried out in natural environments. Actually, due to the complexity of such investigations, research on interspecific competitive interactions among parasitoids has often been characterized by laboratory studies in which hosts are exposed to female parasitoids in various sequences and combinations [8]–[17]. However, experimental laboratory conditions usually differ from field conditions, as the system is simpler, and it is therefore not easy to extrapolate results from laboratory experiments to field populations [18]. In the last years, however, an increasing number of studies investigated interspecific interactions in field and semi-field conditions although just a few number of works focused on egg parasitoids [18]–[20]. Such studies have shed light on the mechanisms explaining how competing parasitoid species can coexist. However, it is still unclear whether multiple biological control agents are more effective than single species in host suppression [21], [22]; nonetheless it has been recently shown that transient dynamics of host-parasitoid communities can play a major role in pest suppression [23].

Surveys on egg parasitoid guilds of herbivorous stink bugs, that are serious pests for a wide number of crops worldwide, have shown that species belonging to the genera *Trissolcus* and *Ooencyrtus* can often naturally co-occur on the same hosts [24]–[28]. In particular, on *Nezara viridula* (L.) eggs, such co-occurrence of parasitoids is widely reported in North America [25], [29]–[31], South America [24], [26], Europe [32] and Japan [33]. In many countries, in order to control stink bug populations, biological control programs based on egg parasitoids have obtained a variable degree of success [25], [31], [34], [35]. Thus, understanding the competitive interactions between *Trissolcus* and *Ooencyrtus* species attacking the same hosts under natural conditions may be useful to improve biological control of such pests. Therefore, we undertook a study to investigate interspecific interactions between *Trissolcus basalis* (Wollaston) and *Ooencyrtus telenomicida* (Vassiliev), two egg parasitoids of *N. viridula* that co-occur in cultivated crops grown in Sicily, Italy. This model system has been extensively investigated under laboratory conditions and information about interspecific extrinsic and intrinsic competition, asymmetrical intraguild parasitism, as well as comparative host location strategies, is available [32], [36]–[40]. Actually, under laboratory conditions, it has been demonstrated that these parasitoid species differ in their host location and larval competitive abilities, with *T. basalis* being more efficient in host location [36]–[38] while *O. telenomicida* largely dominates interspecific larval competition regardless of the order/time interval between ovipositions [32], [39]. Furthermore, *O. telenomicida* has the ability to develop as a facultative hyperparasitoid extending the window of opportunity for parasitism [40]. This background information gained from laboratory experiments can be the basis for better understanding intraguild interactions in the field where such interactions naturally evolve and take place.

In this paper, under semi-field (in out-door mesh cages) and field conditions, we investigated: 1) the seasonal occurrence of competing parasitoid species on sentinel egg masses; 2) the impact achieved by competing species on the shared host on naturally laid egg masses; and 3) the outcome of intraguild interactions under controlled conditions.

## Materials and Methods

### Insect rearing

The *N. viridula* colony was established from insects collected in crop areas around Palermo, South Italy. No specific permits were required for collection of insects. The collection sites were not privately owned or protected in any way and field samplings did not involve endangered or protected species. Bugs were reared in a climatic chamber (24±1°C, 70±5% r.h., and L16:D8 photoperiod), inside wooden cages (50×30×35 cm), with 5 cm diameter mesh-covered holes (200 holes/cm^2^). Immatures and adults were reared in different cages and fed with a diet of seasonal vegetables, cabbages and sunflower seeds. Food was replaced every 2–3 days. Inside adults' cages, paper towels were hung as ovipositional substrate. Egg masses, that were daily collected to prevent adult cannibalism, were used to maintain the colony, to sustain parasitoids rearing and to carry out experiments.

The *T. basalis* and *O. telenomicida* colonies were established from wasps emerging from *N. viridula* egg masses found in crops located around Palermo, Italy. Adult parasitoids of each species were reared, in 85-ml glass tubes, fed with a honey-water solution, and kept in an incubator at 24±1°C, 70±5% r.h., and L16:D8 photoperiod. Two-three times per week, 1–2-day-old egg masses of *N. viridula* were exposed to parasitoids for 48 h, and then stored for insect development under the same climatic conditions. After emergence, male and female parasitoids were kept together to allow mating. All female wasps used for the experiments were 4–5 days old, mated and naïve with respect to oviposition experience. All insect colonies were regularly refreshed with new field materials.

### Seasonal occurrence under natural conditions

In order to monitor the occurrence of *T. basalis* and *O. telenomicida*, sentinel egg masses of *N. viridula* obtained under laboratory conditions were deployed in an experimental field located around Palermo. The research was carried out in 2011 and 2012 from May to October. An experimental field of 0.50 ha was cultivated with tomato, cultivar “Costoluto genovese”. Seedlings were transplanted every year at the beginning of April and arranged at distance of 0.8 m along the row and 1.2 m between rows. Mechanical weed removal and irrigation were carried out when needed. During the growing seasons, natural infestations of *N. viridula* and other minor pests were observed but not treated with pesticides. Using Metylan Normal wallpaper paste glue (Henkel, Dusseldorf, Germany), *N. viridula* sentinel egg masses were artificially assembled to consist of two lateral rows of three hosts each and a central row of four hosts for a total of ten 24 h-old eggs, on 2.5×2.0 cm white cardboards. Weekly, eight cardboards bearing sentinel egg masses were attached to the adaxial surface of the leaves using paperclips on randomly chosen plants that were marked with colored ribbons to better facilitate the recollection. After one week, sentinel egg masses were retrieved, placed individually in 85-ml glass tubes labeled with collection date, taken to laboratory and stored in an incubator (24±1°C, and 70±5% r.h.) until the emergence of parasitoids or the eclosion of stink bug nymphs. Investigations started on May, when no adults of *N. viridula* were yet observed in the field, and finished on October, when all sentinel egg masses were not parasitized for two consecutive weeks.

### Host impact under natural conditions

In order to monitor the host impact achieved by competing parasitoid species, naturally laid host egg masses were sampled in another experimental field also located around Palermo. The research was carried out during the 2011 and 2012 growing seasons from June to September, when natural infestations of *N. viridula* occurred. Experimental tomato field was equal in terms of size, cultivar and agricultural practices to the one described above. The egg mass sampling procedure consisted of a careful visual examination of leaf surfaces of a random selection of plants for ∼3 h. Egg masses discovered were individually placed in 85-ml glass tubes, labeled with collection date, taken to the laboratory and stored in an incubator (24±1°C, and 70±5% r.h.) until the emergence of the parasitoids or the eclosion of stink bug nymphs. Egg masses from which stink bugs or parasitoids have already emerged were also taken into account. Indeed, previous observations had shown that host eggs that yield stink bug nymphs are clearly distinguishable from parasitized ones and that *T. basalis* and *O. telenomicida* are the only egg parasitoid species associated with *N. viridula* in Western Sicily [38]. Furthermore, hosts exploited by these two parasitoid species are also easily distinguishable: *T. basalis* chews a bigger hole from the top of the host egg without releasing meconium whereas *O. telenomicida* chews a smaller hole either from the top or from the side of the host egg leaving the meconium inside (Cusumano, unpublished data). No other parasitoid species was assumed to have emerged from empty egg masses.

### Outcome of intraguild interactions under controlled conditions

In order to evaluate the outcome of intraguild interactions between *T. basalis* and *O. telenomicida*, parasitoids were simultaneously released under semi-field conditions. The research was conducted on July 2012. Sentinel egg masses obtained under laboratory conditions were deployed in an experimental field located at the University of Palermo. The experimental field (100 m^2^) was prepared for seedlings by mechanically removing weeds to prevent competition for space, light and nutrients, and then cultivated with pepper plants, *Capsicum annum* var. “Quadrato d'Asti”. Seedlings were transplanted at the beginning of June and daily watered. Two weeks later, 18 cages of 150×150×70 cm made of wooden frame and mesh fabric net (200 holes/cm^2^) were placed on the field. Each cage included five pepper plants, grown with the following arrangement: one plant in the center of the cage and four plants, which were equally distant 50 cm from the central one, near to the cage corners. When the pepper plants reached about 45 cm of height, a 10-eggs sentinel egg mass of *N. viridula*, prepared as described above, was attached with paperclips to a medium sized leaf of each of the four lateral pepper plants. Parasitoids were then gently released on the central plant according to the following combinations: (1) single release of 10 females of *T. basalis*; (2) single release of 10 females of *O. telenomicida*; (3) simultaneous release of 10 females of both species. After a week, *N. viridula* egg masses were recollected and stored singly in 85-ml glass tubes into an incubator (24±1°C, 70±5% r.h., and L16:D8 photoperiod) until the emergence of the parasitoids or the eclosion of stink bug nymphs. For each combination, six replicates were performed using a completely randomized design.

### Collection data

For both field and semi-field investigations, insect emergences from stored egg masses were used to compute the following efficiency indexes proposed by Bin and Vinson [41]: 1) *host location*  =  number of egg masses from which at least one parasitoid emerged divided by the total number of sampled egg masses; 2) *host exploitation*  =  number of individually parasitized eggs divided by the total number of eggs from the located egg masses; 3) *host impact*  =  number of individually parasitized eggs divided by the total number of sampled eggs.

The host location index assesses the parasitoids' searching ability whereas the host exploitation index estimates the parasitoids' reproductive capacity once an egg mass has been located. The host impact, that takes into account all collected egg masses, either parasitized or unparasitized, assesses the overall parasitoids' efficiency as biological control agents. In order to better assess interspecific interactions, data were distinguished according to single (*T. basalis*; *O. telenomicida*) and concurrent (*T. basalis + O. telenomicida*) emergence of the egg parasitoid species from the same egg mass.

### Statistical analysis

Host location data from naturally laid egg masses were analyzed with a contingency table χ^2^ using Bonferroni correction to find significant differences. Such analysis was performed by pooling together data of 2011 and 2012 because there was no statistical difference between both years (χ^2^ = 0.55, df = 3, *P* = 0.907). Data of host exploitation and host impact from naturally laid egg masses were analyzed, separately for each year, with a logistic regression which is a generalized linear model (GLM) designed for modeling binomial data with the logit link function. In this case, for host exploitation, the number of individually parasitized eggs and the non parasitized eggs from located egg masses were used as binomial dependent variables. For host impact, the number of individually parasitized eggs and the total number of non parasitized eggs from all egg masses were used as binomial dependent variables. In order to compare species abilities in locating and exploiting naturally laid egg masses, an additional analysis was conducted by pooling together all data from a single species regardless if parasitoids emerged singly or in egg mass also attacked by the interspecific competitor. More accurately, a logistic regression with year, species, egg mass size as factors and egg mass size × species interaction, was performed to analyze host location whereas a GLM for Poisson distribution using the log link function was performed to analyze host exploitation data.

Data from semi-field experiments were also analyzed with a logistic regression using each egg mass as a different replicate unit. Logistic regression was followed by Tukey contrast for multiple comparisons. To assess the possible effect of interspecific interactions on the ability of parasitoids to impact the host population, the observed levels of *N. viridula* mortality in the multiple species release treatment (*T. basalis* + *O. telenomicida*) were compared to expected levels of *N. viridula* mortality calculated using data from the single species release treatments only (*T. basalis*; *O. telenomicida*). If interspecific interactions among parasitoid species have no effect on the host population (parasitoids have independent effects), the levels of host mortality should follow a multiplicative risk model [18], [42]:
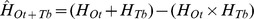
where 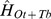 is the expected host mortality by parasitoids *T. basalis* and *O. telenomicida* together, 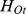 the observed host mortality by *O. telenomicida* alone and 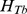 the observed host mortality by *T. basalis* alone. The observed and expected levels of *N. viridula* mortality were compared, for each replicate, using simple χ^2^ tests. Since data were independent, results of each of *n* replicate were combined using an Omnibus test to globally test the null hypothesis that there is no difference between observed and expected values leading to χ^2^ with 2*n* df [43], [44]. Significant differences corresponding to a higher expected levels of host suppression compared to the observed levels would indicate a negative effect of interspecific competition on host population suppression. All statistical analyses were performed with the R 2.14.1software [45] and multiple comparisons were done with the *multcomp* package [46].

## Results

### Seasonal occurrence under natural conditions

**Figure 1:**
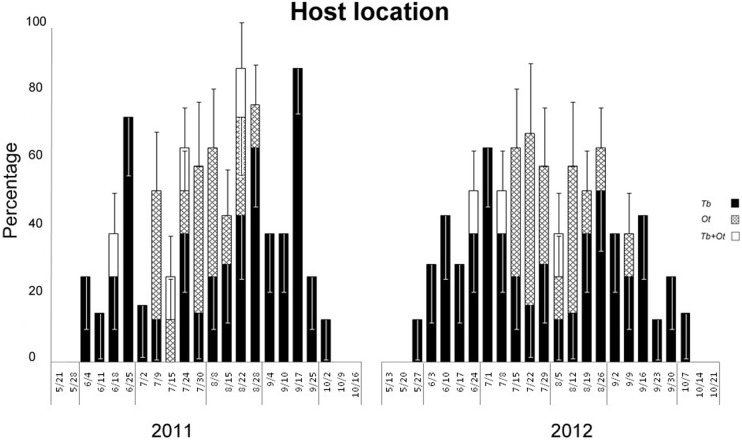
Seasonal occurrence of parasitoid species (% ± SE) recorded from sentinel egg masses in 2011 and 2012 under field conditions. Data are reported in terms of *host location*  =  number of egg masses from which at least one parasitoid emerged divided by the total number of recollected egg masses. Black, cross and white bars indicate egg masses discovered by *T. basalis* only (Tb), by *O. telenomicida* only (Ot) and by both species (Tb+Ot), respectively.

In 2011, successful parasitism by *T. basalis* was first recorded on sentinel eggs deployed on June 4 and this species always occurred until October 2 (fig. 1). Compared to *T. basalis*, occurrence of *O. telenomicida* alone was recorded for a shorter time during the season, from July 9 until August 28. Co-occurrence of both parasitoid species from one egg mass was also recorded in four sampling dates (June 18, July 15, July 24 and August 22). A similar seasonal pattern was reported in 2012 when *T. basalis* was again recorded before *O. telenomicida* and for a longer time throughout the season. Actually, emergence of *T. basalis* was first recorded on sentinel eggs placed in the field on May 27 and this species always occurred until October 7. Occurrence of *O. telenomicida* only was recorded for a shorter time during the season, from July 15 until September 9. Emergence of both parasitoid species from one egg mass was also recorded in three sampling dates (June 24, July 8 and August 5).

### Host impact of parasitoid species under natural conditions

**Figure 2:**
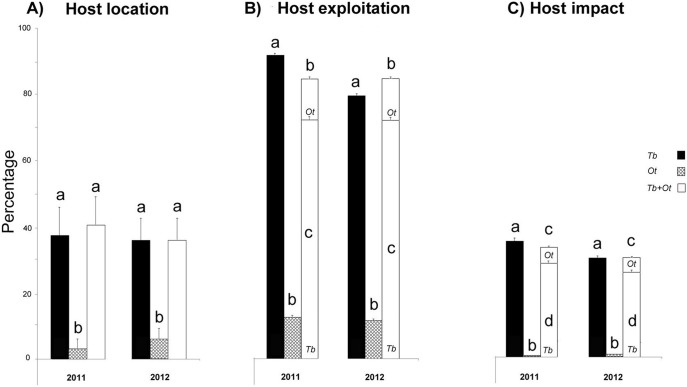
Parasitism data (% + SE) recorded from naturally laid egg masses in both 2011 and 2012 under field conditions. All indexes are distinguished according to single or concurrent emergence of the egg parasitoid species from the same egg mass. (A) *host location*  =  number of egg masses from which at least one parasitoid emerged divided by the total number of egg masses. (B) *host exploitation*  =  number of individually parasitized eggs divided by the total number of eggs from the egg masses located by the wasps and (C) *host impact*  =  number of individually parasitized eggs divided by the total number of sampled eggs. In (B) and (C) black, cross and white bars indicate the proportion of individual eggs exploited from egg masses located by *T. basalis* only (Tb), by *O. telenomicida* only (Ot) and by both species (Tb+Ot), respectively. Different letters indicate significantly different percentages within the same year and index (C) (GLM, P<0.05).

In both 2011 and 2012, a total of 32 and 50 egg masses naturally laid by *N. viridula* were collected, respectively. The proportion of egg masses that were unparasitized or located by parasitoid species was not significantly different between 2011 and 2012 (χ^2^ = 0.55, df = 1, *P* = 0.907) but host location efficiency was affected by parasitoid emergence combination (*T. basalis*, *O. telenomicida*, *T. basalis + O. telenomicida*). More accurately, host location was not significantly different between *T. basalis versus T. basalis + O. telenomicida* (χ^2^ = 0.03, df = 1, *P*>0.05) but it was significantly different between *T. basalis* versus *O. telenomicida* (χ^2^ = 25.08, df = 1, *P*<0.001) as well as between *O. telenomicida* versus *T. basalis + O. telenomicida* (χ^2^ = 26.48, df = 1, *P*<0.001) (fig. 2A). In both 2011 and 2012, a total of 2272 and 3480 individual eggs were exploited by parasitoids from the located egg masses, respectively. The proportion of individual host eggs that were exploited by parasitoid species was significantly affected by the emergence combinations (GLM: χ^2^ = 3943.4, df = 7, *P*<0.001) (fig. 2B). Regardless of the year, host exploitation efficiency by *T. basalis* was reduced when this species exploited eggs in single *versus* concurrent exploitation whereas the host exploitation efficiency by *O. telenomicida* was not significantly affected (Tukey test, *P*<0.05).In both 2011 and 2012, a total of 2810 and 4371 individual eggs were sampled, respectively and the host impact index was significantly affected by the emergence combinations (GLM: χ^2^ = 4725.9, df = 7, *P*<0.001) (fig. 2C). Regardless of the year, the impact achieved by *T. basalis* and by *O. telenomicida* was significantly different when species exploited eggs in single *versus* concurrent exploitative conditions (Tukey test, *P*<0.05).

On naturally laid egg masses, a significant effect of the species (GLM: χ^2^ = 17.36, df = 1, *P*<0.001) was found on the host location efficiency, whereas the effect of the year (GLM: χ^2^ = 0.25, df = 1, *P* = 0.617), of the eggs mass size (GLM: χ^2^ = 0.00, df = 1, *P* = 1.000) and of the species × eggs mass size interaction (GLM: χ^2^ = 0.30, df = 1, *P* = 0.584) were all not significant. However, a significant effect of the species (GLM: χ^2^ = 1936.92, df = 1, *P*<0.001), of the egg mass size (GLM: χ^2^ = 288.21, df = 1, *P*<0.001) and of species × egg mass size interaction (GLM: χ^2^ = 65.95, df = 1, *P*<0.001) was found on host exploitation.

### Outcome of intraguild interactions under controlled conditions

**Figure 3:**
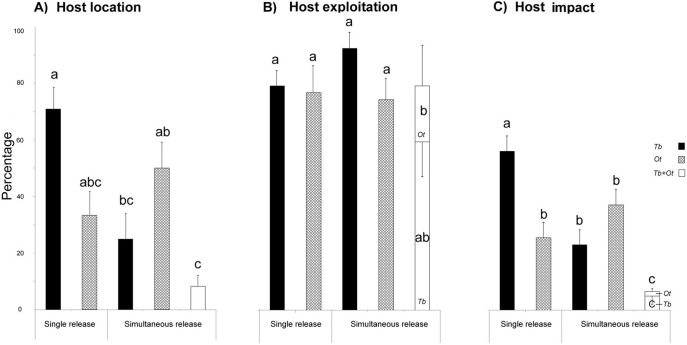
Parasitism data (% + SE) obtained when species were released singly or simultaneously under semi-field conditions. All indexes are distinguished according to single or concurrent emergence of the egg parasitoid species from the same egg mass. (A) *host location*  =  number of egg masses from which at least one parasitoid emerged divided by the total number of egg masses. (B) *host exploitation*  =  number of individually parasitized eggs divided by the total number of eggs from the located egg mass and (C) *host impact*  =  number of individually parasitized eggs divided by the total number of sampled eggs. In (B) and (C) black, cross and white bars indicate the proportion of individual eggs exploited by parasitoids from egg mass located by *T. basalis* only (Tb), by *O. telenomicida* only (Ot) and by both species (Tb+Ot), respectively. Different letters indicate significantly different percentages within the same index (GLM, P<0.05).

The host location index of parasitoid species was significantly affected by the release and emergence combinations (GLM: χ^2^ = 25.22, df = 4, *P*<0.001) (fig. 3A). The host location index of *T. basalis* only was significantly different in single *versus* simultaneous release whereas the host location index of *O. telenomicida* was not affected (Tukey test, *P*<0.05).

The proportion of individual host eggs that were exploited by parasitoid species was also significantly affected by the release and emergence combinations (GLM: χ^2^ = 22.37, df = 5, *P*<0.001) (fig. 3B). Host exploitation efficiency of *T. basalis* was not significantly different when this species exploited eggs in different release and emergence combinations (single release, single emergence in simultaneous release and concurrent exploitation), whereas the host exploitation efficiency of *O. telenomicida* decreased in condition of concurrent exploitation compared with single release or single emergence in simultaneous release (Tukey test, *P*<0.05).

Similarly to host location and exploitation, the host impact index was also significantly affected by the release and exploitation combinations (GLM: χ^2^ = 151.28, df = 5, *P*<0.001) (fig. 3C). The impact of *T. basalis* was significantly higher for single release, intermediate for single emergence in simultaneous release and very low in conditions of concurrent exploitation (Tukey test, *P*<0.05), whereas the impact of *O. telenomicida* was not statistically different between conditions of single release *versus* single exploitation in simultaneous release but it was much lower for concurrent exploitation in simultaneous release (Tukey test, *P*<0.05).

Regardless of the fact that data were compared separately for each replicate or combined together, no significant differences were found between observed and expected levels of *N. viridula* mortality inflicted by parasitoids in the two species combination (Ĥ_Ot+Tb_ = 65.21%; H_Ot+Tb_ = 67.50%) (χ^2^ = 2.21, df = 12, *P*>0.05).

## Discussion

Egg parasitoids are organisms of particular interest for biological control due to their ability to kill insect pests before the crop-feeding stage [47]. In the perspective of biological control of stink bugs, an efficient egg parasitoid species must be characterized not only by a high host location index, *i.e.* a high ability to discover host egg masses, but also by a high exploitation efficiency in order to parasitize all the available eggs within an egg mass [41]. Parasitoid species characterized by high host location and host exploitation abilities have consequently a high host impact index and are most likely interesting for biological control programs [48]. However, such indexes can be affected by intraguild interactions due to the interference on reproductive success of each species [49], [50].In the field, competitive interactions among parasitoids determine natural community structure and dynamics in the plant–herbivore–parasitoid systems, for example, by causing local displacement of inferior species or niche separation, and can play a relevant role in modifying the efficacy of parasitoids in biological control programs [51]. In our system, although *T. basalis* and *O. telenomicida* compete for *N. viridula* egg mass, they coexist under field conditions, likely by adopting different strategies to exploit the shared resource.

The results from semi-field and field investigations, coupled with previous findings obtained under laboratory conditions aimed at better understanding intraguild interactions between egg parasitoids of a true bug. In particular, semi-field experiments investigated interspecific interactions under manipulative conditions of simultaneous species release in order to obtain high competition for hosts. Our results from sentinel egg masses placed in the field indicated a different pattern of occurrence of competing parasitoid species. In both years, *T. basalis* occurred in late May-early June and it was found parasitizing host eggs until late September-early October whereas *O. telenomicida* occurred mainly in July-August. Interestingly, similar temporal patterns of occurrence of stink bug egg parasitoid species have been found in other case studies confirming that *Ooencyrtus* species are more abundant later in the season whereas competing *Trissolcus* and *Gryon* species are commonly found earlier [27], [28], [52]. The effects of abiotic conditions on the timing of occurrence of egg parasitoid guilds have already been reported, suggesting a role played by species' differences in cold tolerance and/or humidity requirements: for example, *Trissolcus brochymenae* ( = *murgantiae*) Ashmead is more cold-tolerant than its competitor *Ooencyrtus johnsonii* (Howard) [53] whereas *Gryon japonicum* (Ashmead) is likely to perform better with lower humidity than its competitor *Ooencyrtus nezarae* Ishii [52]. The effects of weather conditions, especially cold tolerance, on the timing of occurrence of competing parasitoid species likely played a role also in our study system but further investigations are required in order to test whether *T. basalis* is able to parasitize host eggs under colder temperature conditions compared to *O. telenomicida*. In addition, these parasitoid species may have different overwintering requirements: it is possible that *T. basalis* completes overwintering period before *O. telenomicida* and, consequently, the former can occur earlier in the field. From a biological control perspective, the demonstration that the two species are active at different times of the season might suggest that the combination of the two parasitoids is likely to outcompete the level of control generated by each species alone. Such hypothesis could be supported by the results from the multiplicative risk model which indicated that host mortality when the two parasitoids are released together is not significant different than that one expected under independent action of the two species. However, short- and long-term population investigations taking into account host-parasitoid dynamics are required to confirm the results presented in this study.

Results obtained from naturally laid egg masses highlight a higher host location index and, thus, a superior host finding ability of *T. basalis* compared to *O. telenomicida*. These findings confirm previous laboratory data with Y-tube and open area bioassays. Indeed, *T. basalis* is known to exploit volatile oviposition-induced synomones, volatile cues from virgin males and preovipositing females, and contact kairomones in the host footprints [36], [37], [54]–[56], while *O. telenomicida* uses mainly volatile cues produced by host virgin males [38]. Interestingly, *T. basalis* apparently exploits not only more cues than *O. telenomicida* but also the more reliable ones, i.e., cues that are highly associated with the host presence such as synomones-induced volatiles. Once an egg mass has been located, *T. basalis* is able to exploit a high proportion of the available host eggs whereas *O. telenomicida* is much less efficient and achieves similar efficiency regardless of single and concurrent exploitation. Such higher level of host exploitation by *T. basalis* has also been documented [25], [31], [48], [57].

The main factors that are likely to affect species differences in host exploitation are reproductive abilities, such as egg load and total lifetime fecundity, which are known to be higher in *T. basalis* compared to *O. telenomicida* (average egg loads: *T. basalis*  = 76.2 eggs; *O. telenomicida*  = 24.2 eggs, Cusumano, unpublished data). Such differences in terms of host exploitation are not evident in our semi-field investigations, where both species demonstrated similar performance in condition of single or simultaneous release, probably because both parasitoid species have a sufficient egg load for exploiting small-sized host egg masses. In these experiments, egg masses artificially assembled with 10 eggs only have been used to enhance interspecific competitive interactions, like in previous laboratory experiments [32], [40]. Therefore, under natural conditions, *T. basalis*, which is characterized by superior host location and host exploitation abilities than *O. telenomicida*, achieved a higher impact on *N. viridula* egg masses. As a consequence, chances that *O. telenomicida* females find unparasitized egg masses could remain quite low.

It has to be noted that the high proportion of egg masses located by both parasitoid species in the field shows that *O. telenomicida* has most likely evolved some strategies to overcome its inferior abilities to locate hosts. Actually, by being superior under the conditions of interspecific larval competition, and by being able to act as facultative hyperparasitoid, *O. telenomicida* can extend its window of opportunity to exploit host eggs for an additional 6–7 days. Indeed, *O. telenomicida* can successfully develop on host eggs laid by *N. viridula* females up to 10 days before, while *T. basalis* can only successfully develop on unparasitized host eggs that are <4 days old [40]. Hence, the coexistence between these two species represents an example of counterbalanced competition [58]. Counterbalanced competition suggests that coexistence among parasitoids attacking the same host can be possible when one species, in our case *O. telenomicida*, dominates the intrinsic competition, while the other, in our case *T. basalis*, fills the gap at the extrinsic level by having higher host finding or dispersal efficacies.

The advantage of *O. telenomicida* over *T*. *basalis* under the conditions of interspecific larval competition is clearly showed by our results obtained in semi-field experiments. Under such conditions, *O. telenomicida* performs similarly regardless if it was released alone or in combination. On the contrary, *T. basalis* achieves higher host location efficiency and host impact when released alone, while it suffers from *O. telenomicida* competition when the two species are released in combination. Moreover, the proportion of egg masses located by both species under semi-field conditions is lower than in natural fields. Results from semi-field are in apparent contrast with those obtained under natural conditions. However, this can be explained taking into account the small size of host egg masses used in the semi-field experiments. Under such conditions, competition for hosts is particularly severe and *T. basalis* can be excluded by *O. telenomicida*, due to the very low reproductive success of the former species when ovipositing into host eggs that are also attacked by its competitor, as was showed previously under laboratory conditions [32], [40]. Consequently, it is possible that *T. basalis* has oviposited into multiparasitized host eggs yielding *O. telenomicida* offspring and, as a result, the host location index of the former species could have been reduced. Competitive exclusion of *Trissolcus* species by *Ooencyrtus* species under laboratory conditions has also been demonstrated between *T. brochymenae* and *O. johnsonii* on egg masses of the harlequin bug *Murgantia histrionica* (Hahn), which naturally consist of 12 eggs each [53]. However, naturally laid egg masses of *N. viridula* are usually much bigger [59]. For example, in our 2-year study, the average size of natural egg masses collected was 87.57±2.05 eggs. Therefore, it is possible to speculate that, under natural conditions, multiparasitism in every host egg is probably less likely to happen and, consequently, competitive exclusion rarely occurred.

Hence, on naturally laid egg mass, it is possible that interspecific interactions are less strong due to the egg mass size and *T. basalis* can compensate for the progeny loss caused by *O. telenomicida* thanks to its superior extrinsic abilities in host location and exploitation. Since data on natural egg masses are observational (i.e., not experimentally manipulated), and thus not replicable, further experiments in order to assess the role of egg mass size and parasitoid reproductive capacities on intraguild interactions under controlled conditions are required.
